# 2226. Trough- Versus AUC-Based Vancomycin Monitoring for Treatment of Acute Pulmonary Exacerbations of Adult Cystic Fibrosis Patients

**DOI:** 10.1093/ofid/ofad500.1848

**Published:** 2023-11-27

**Authors:** Darrell Smith, Marguerite Monogue, James Sanders

**Affiliations:** UT Southwestern Medical Center, Dallas, Texas; University of Texas Southwestern Medical Center, Dallas, TX; UT Southwestern Medical Center, Dallas, Texas

## Abstract

**Background:**

Therapeutic drug monitoring (TDM) for IV vancomycin (VAN) in adults with cystic fibrosis (CF) historically has utilized trough concentrations. Recent VAN TDM guidelines recommend area under the curve (AUC)-monitoring to reduce the risk of vancomycin-induced kidney injury. However, limited data is available in adult CF patients to support this practice. The aim of the study was to determine the safety and efficacy of VAN AUC-monitoring in adult CF patients.

**Methods:**

This single-center, retrospective, observational cohort study included adult CF patients admitted from July 1, 2017 to July 1, 2022 with an acute pulmonary exacerbation that received VAN for at least 72 hours with available VAN plasma concentrations for TDM. Eligible patients with multiple hospital admissions during the study period were incorporated as separate encounters. The primary outcome was incidence of acute kidney injury (AKI). A subgroup efficacy analysis was performed in patients with methicillin-resistant *S. aureus* (MRSA) in the sputum 3 months prior to or 3 weeks after hospitalization.

**Results:**

One hundred forty-three patients were included in the study [AUC cohort (n=39) and trough cohort (n=104)]. Concurrent nephrotoxins was more common in the AUC cohort than in the trough cohort (97% vs 81%; p = 0.01), but rate of AKI was similar (7.7% vs 10.6%, respectively; p = 0.76). No significant differences were seen in achievement of TDM goal, incidence of supratherapeutic exposure or vancomycin accumulation, duration of vancomycin, length of stay, or number of vancomycin concentrations. AUC monitoring was associated with earlier achievement of TDM goal [median 0 days (0-2) vs 2 days (0-4); p < 0.01], lower total daily doses to achieve TDM goal [38.6 ±13 mg/kg/day vs 58.4 ±18.7mg/kg/day; p < 0.01], and fewer regimen changes [median 1 change (0-2) vs 2 changes (1-3); p < 0.01] compared to trough monitoring. In patients with MRSA, pulmonary function recovery, readmission, and mortality was similar.

**Figure:**
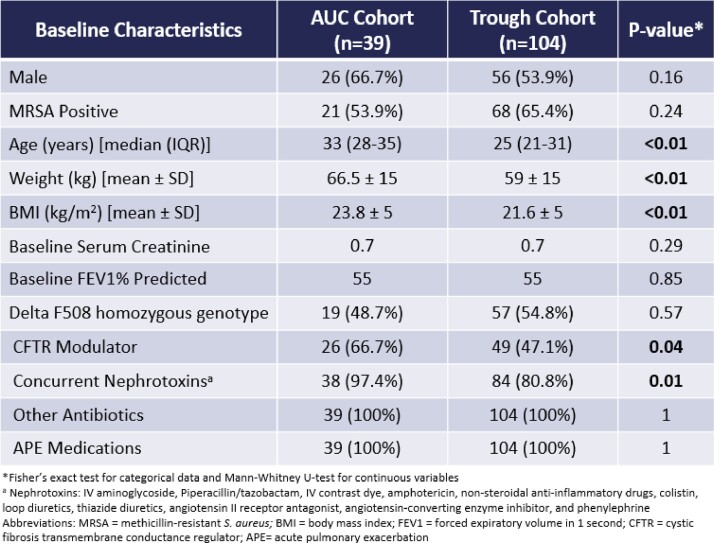

**Figure:**
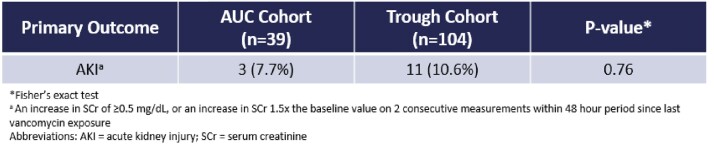

**Figure:**
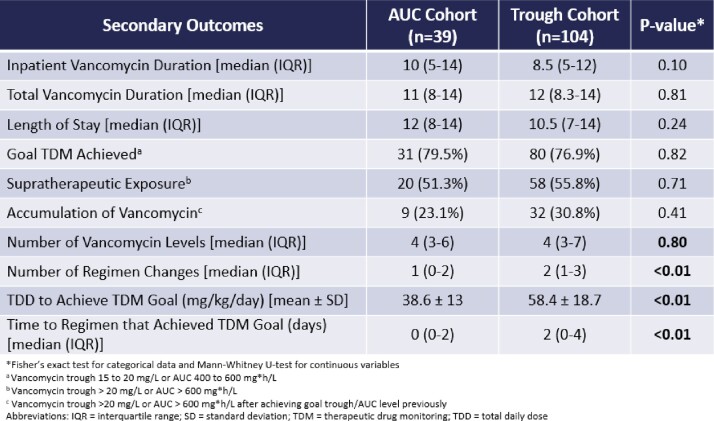

**Conclusion:**

In adult CF patients, the rates of AKI were similar between VAN TDM methods, but VAN AUC monitoring resulted in a therapeutic regimen sooner at lower total daily VAN doses and fewer number of regimen changes without significantly increasing the number of concentrations compared to trough monitoring.

**Disclosures:**

**James Sanders, PhD, PharmD**, Merck & Co., Inc.: Grant/Research Support|Shionogi Inc.: Grant/Research Support

